# Anæmia

**Published:** 1901-04-27

**Authors:** 


### ANÆMIA.

The discussion on anæmia which took place at the last meeting of the Balneological and Climatological Society, tended to show how far we have drifted away from the old notion that chlorosis, or indeed anæmia of any kind, is a primary disease, standing by itself, and to be cured by remedies directed to the blood condition alone. When, however, we come to inquire what are the causes of anæmia, we find great differences of views, and are led at once to what is probably the correct conclusion—namely, that the blood, circulating as it does through all the tissues of the body, and coming in contact with whatever of disease there may be in any part, is liable to be affected injuriously in many ways, the results of all of which we call "anæmia." The rapidity with which variations in the state of the blood take place in the course of certain acute diseases, is sufficient to show how susceptible it is and how quickly it recuperates, and we are almost driven to the conclusion that a persistent anæmia, of whatever type, indicates a persistence of some disordered condition, often, no doubt, a persistent toxæmia. Dr. Haig points to uric acid, and regards anæmia as an almost universal disease in eaters of flesh and drinkers of tea. Dr. St. Clair Thomson points to hidden sources of chronic suppuration in the ear, nose, pharynx, and bronchi as giving rise to secondary septic anæmias. Dr. Bezly Thorne entertains no doubt that in cases of chlorosis or the more persistent form of anæmia, the pathological basis of the condition is a gastro-duodenal catarrh, leading to interference with the flow of the biliary and pancreatic juices, and inducing gastro-intestinal fermentative decomposition and consequent auto-intoxication. Thus, so far from regarding iron as a panacea, he asserted that he had seen more benefit arise in a fortnight from iodides, combined with bismuth and alkalies, and similar eliminative treatment, than he had from months or years of the administration of iron, and he attributed the "superlative benefit" of the Nauheim methods in anæmia very largely to the free diuresis which that treatment almost invariably rapidly induced. Other speakers pointed to the benefits derived from the use of the eliminative mild sulphur at Harrogate in the treatment of anæmia, as showing that elimination rather than iron was required in anæmia. In regard to this we may refer to the opinion now held by many that of all the iron so freely given in many cases, the amount absorbed is absolutely infinitesimal, and that the good which large doses of iron really appear to do in some cases, arises from its action as an intestinal antiseptic. The point of interest arising from this discussion is that in every case of anæmia the most careful search must be made for some septic, dyspeptic, or dietetic cause of toxæmia, and that, useful as iron and fresh air and change may be, this persisting toxic influence, whatever it may be, must be removed.

